# Major Intrahepatic Veno-Venous Fistula after Fontan Operation Treated by Transcatheter Implantation of Amplatzer Septal Occluder through Internal Jugular Vein

**DOI:** 10.5935/1678-9741.20160020

**Published:** 2016

**Authors:** Alper Guzeltas, Ibrahim Cansaran Tanidir, Murat Saygi

**Affiliations:** 1 Mehmet Akif Ersoy, Thoracic and Cardiovascular Surgery Center and Research Hospital, Istanbul, Turkey

**Keywords:** Heart defects, congenital, Child, Fontan Procedure

## Abstract

Six months after undergoing a Fontan operation, a 7-year-old boy with right atrial isomerism and a single functional ventricle was admitted to our emergency department with cyanosis. Emergency cardiac catheterization revealed a large veno-venous fistula that began in a left hepatic vein, connected to the left accessory hepatic veins, and drained into the common atrium, resulting in desaturation. The fistula was occluded proximally with an Amplatzer septal occluder, with satisfying results; the patient's systemic arterial saturation decreased during his hospital stay. Three weeks after the first intervention, a second procedure was performed to retrieve the first device and to close the fistula distally. Multiple attempts with different types of gooseneck snares and a bioptome catheter failed to retrieve the first device, so a telescopic method was used to re-screw it. Using a Mullins long sheath and delivery sheath, the delivery cable was manipulated to fit into the slot of the end screw, and the cable was rotated gently in a clockwise direction to re-screw the device. Then, another Amplatzer septal occluder was placed at the distal end of the fistula. In conclusion, distal transcatheter occlusion of intrahepatic veno-venous fistulas might lead to better clinical outcomes in selected patients. Amplatzer septal occluder device can be retrieve without any complication within three weeks.

**Table:** 

Abbreviations, acronyms & symbols	
ASO	= Amplatzer septal occluder
TAPVC	= Total anomalous pulmonary venous connection

## INTRODUCTION

Complex cyanotic congenital cardiac diseases are sometimes not suitable for biventricular repair^[1,2]^. The Fontan operation is a good palliation used in single ventricle physiology to place the systemic and pulmonary circulations in series. Unfortunately, unexpected cyanosis can occur that is often caused by decompressing veno-venous collaterals, originating from systemic veins and draining into the pulmonary veins or the pulmonary venous atrium^[3]^. In patients with visceral heterotaxy, there are various types of systemic venous connections, such as the drainage of hepatic veins independently of the inferior vena cava directly into the common atrium^[1]^. These types of hepatic venous malformations are seen in 8% of heterotaxy patients undergoing Fontan procedures^[4]^. Veno-venous collaterals from the hepatic system to the pulmonary venous atrium have been described in previous studies, along with techniques to eliminate them^[1-6]^. Herein, we describe the transcatheter treatment of a patient with right atrial isomerism and a large venous fistula from a left hepatic vein connected to the left accessory hepatic veins and drained to the pulmonary venous atrium, causing desaturation. Ethics board approval is not required for case reports in our institution.

## CASE

**Fig. 1:**
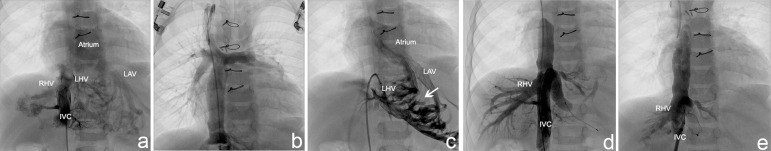
A: Angiogram of the inferior vena cava: a veno-venous malformation between the inferior vena cava and the atrium is detected with a distal collector. B: Angiogram of the superior vena cava revealing poor filling of the pulmonary arteries and rapid retrograde filling of the left inferior caval vein. C: Left hepatic vein originating from the inferior vena cava, dividing into multiple sinusoidal channels in the liver, joining with the left accessory veins, and draining into the pulmonary venous atrium. D: Occlusion test with a balloon in the distal collector under the common atrium. E: Atrial septal device that performed a nearly complete occlusion of the veno-venous malformation. RHV=right hepatic vein; LHV=left hepatic vein; IVC=inferior vena cava; LAV=left accessory veins. Arrow shows multiple sinusoidal channels.

A 7-year-old male patient diagnosed with right atrial isomerism, unbalanced complete atrioventricular septal defect, double outlet right ventricle, pulmonary stenosis, and nonobstructive total anomalous pulmonary venous connection (TAPVC) draining into the left superior vena cava was referred to our clinic. His systemic oxygen saturation was 65-70%. Diagnostic catheterization prior to undergoing a Fontan operation consisted of angiography and pressure measurements in the pulmonary arteries. A Fontan operation using a 16 mm extracardiac conduit was performed, along with TAPVC repair, and the patient's systemic oxygen saturation increased to 90% after surgery. During outpatient follow-up, the patient's saturation was in the high 80s, but six months after surgery, he was admitted to the emergency department with cardiac arrest. He was intubated, and despite with adequate ventilation and 100% FiO_2_, his systemic oxygen saturation was in the lower 30s, and emergency cardiac catheterization was performed on the same day. Mean Fontan pressure was 20 mmHg, and bilateral pulmonary venous return appeared appropriate, with no suggestion of significant pulmonary arteriovenous malformations; no fenestration was determined. However, an inferior vena cava angiogram revealed that a left hepatic vein originating from the inferior vena cava divided into multiple sinusoidal channels in the liver, joining together with the left accessory hepatic veins and draining into the pulmonary venous atrium. Angiographic visualization of the lateral tunnel and superior vena cava revealed poor filling of the pulmonary arteries and rapid retrograde filling of the left inferior caval vein (Figures 1A, 1B and 1C), creating a deviation in blood flow from the systemic venous return to the common atrium.

An occlusion test was performed, and after 10 min of inflation, the inferior vena cava pressure decreased to 17 mmHg. An angiogram conducted during the occlusion test showed better flow to the pulmonary arteries, and systemic oxygen saturation increased from 30% to 85% (with 100% FiO_2_). The fistula was closed proximally with a 15 mm Amplatzer septal occluder (ASO) (AGA Medical, Golden Valley, MN) (Figure 1D, 1E and Movie-1).

The patient was extubated two days after catheterization. His saturation was in the high 85s, but three weeks after the intervention, while still in the hospital, his saturation level decreased to 75-80%. An echocardiograph showed no residual flow through the device, which was attributed to the accessory left hepatic veins returning to the atrium. Another intervention was planned for a later date, either transcatheter or surgical, to retrieve the first device and close the major intrahepatic venovenous fistulas distally.

**Fig. 2:**
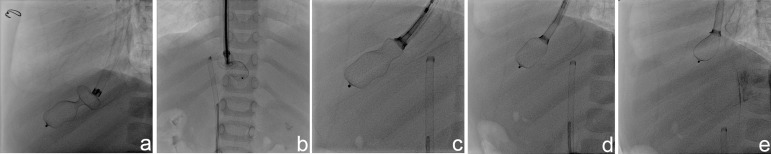
A: 14F Mullins long sheath placed over the end screw of the ASO. B and C: Telescopic system introduced to the distal tip of the ASO. D and E: ASO device retrieved into the Mullins long sheath.

The patient was intubated prior to the procedure, and 6F sheaths were placed into the right jugular vein and femoral vein. Multiple attempts with different types of gooseneck snares and a bioptome catheter failed to retrieve the first device. Re-screwing the device was then considered, as the position of the device appeared favorable for the retrieval. A telescopic method was used to re-screw the device. A 14F Mullins sheath (USCI, Billerica, MA) was placed over the end screw of the ASO and the tip of the Mullins sheath's dilatator was cut distally, large enough to enable a 7F JR4 guiding catheter to be advanced through it. Then, the dilatator was placed at the end screw of the atrial septal defect device through the 14F Mullins sheath, and the 7F JR4 guiding catheter was advanced through until it met the end screw of the ASO. Then, the delivery cable was advanced inside the sheath close to the end screw and it was manipulated to fit into the slot of the end screw. The cable was rotated gently in a clockwise direction to re-screw the device, and the device was retrieved, using the 7F JR4 guiding sheath and 14F Mullins sheath's dilatator in the 14F primary sheath (Figure 2 and Movie-2).

**Fig. 3:**
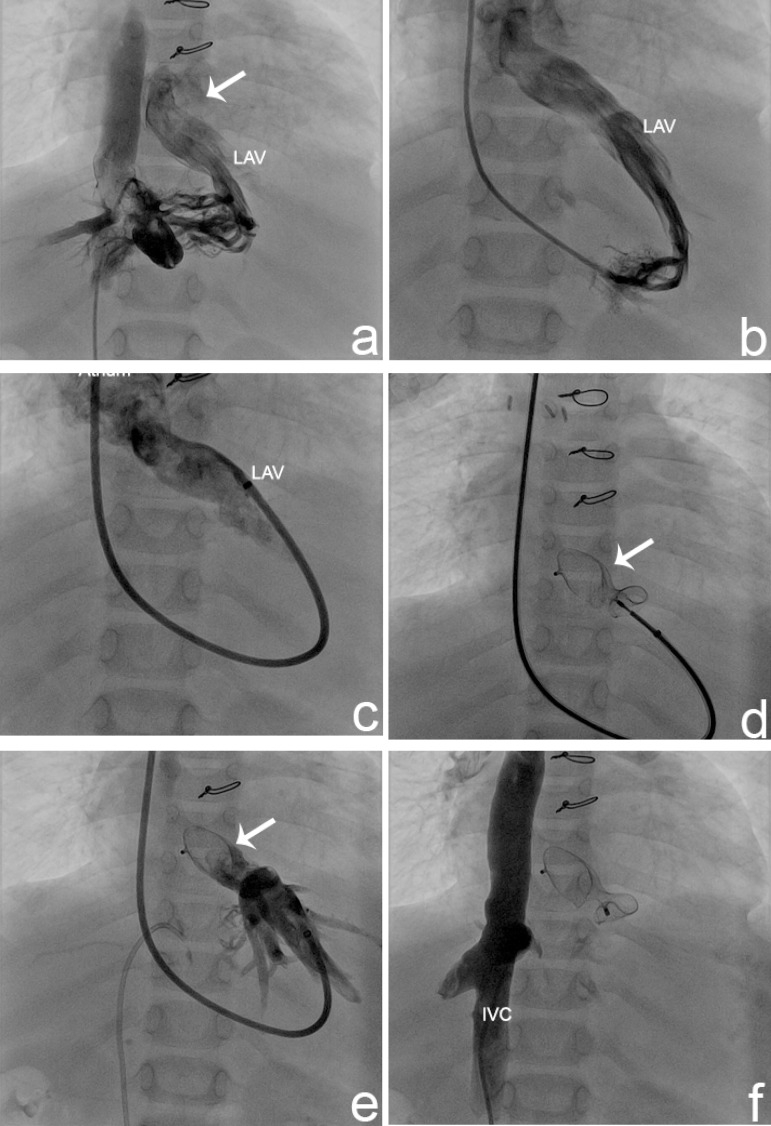
A: Angiogram of the left hepatic vein. B: Angiogram of the left accessory hepatic vein. C: 7F Cook Ansel guiding sheath advanced to the distal part of the left accessory hepatic vein. D: 16 mm ASO opened the atrium-hepatic vein junction. E: Angiogram of the left accessory hepatic vein after the device was released. F: No residual flow into the left hepatic vein and better flow to the pulmonary arteries. IVC= inferior vena cava; LAV= left accessory veins. Arrow shows atrium-hepatic vein junction.

A 5F hydrophilic catheter and a guidewire were then advanced from the right jugular vein through the left hepatic vein and to the right atrium. A stiff exchange wire was placed into the atrium through the hydrophilic catheter. A 7F Ansel guiding sheath (Cook, Bloomington, IN) was advanced into the right atrium, and the veno-venous fistula was closed distally with a 16 mm ASO (Figure 3 and Movie-2). The patient was extubated one day after the procedure, and systemic oxygen saturation was 92-93%. Coumadin was initiated after the procedure, and the patient was discharged one week later. Four months after the procedure, the patient's saturation was 92-94%.

## DISCUSSION

After the Fontan operation, there is an obligatory pressure gradient from the systemic veins to the pulmonary veins. This physiology might stimulate the enlargement of rudimentary embryologic connections from the systemic veins to the pulmonary veins or the pulmonary venous atrium, which can result in decompressing veno-venous collaterals and causing desaturation. These collaterals can include left superior vena cava to coronary sinus, hemiazygos vein entering the left superior vena cava below the level of a previous ligation, systemic to pulmonary vein connections, large coronary veins to the pulmonary venous atrium, and hepatic veins to the pulmonary venous atrium^[3,5]^.

One or more hepatic veins are left connected to the pulmonary venous atrium (called partial exclusion of hepatic vein) or the inferior vena cava is partitioned, with the rationale of minimizing hepatic venous congestion, thus decreasing Fontan pathway volume and pressure load and decreasing serous effusions. This method is advocated as a fenestration technique that leads to smooth postoperative recovery^[2,4-6]^. However, intrahepatic connections can develop between the inferior vena cava and the excluded hepatic vein and result in significant desaturation^[5]^. Proximal transcatheter closure of these fistulas might appear to be easier, but it excludes the all left hepatic vein from systemic venous circulation. The use of this technique should probably be restricted to patients with multiple suprahepatic veins that drain into the common atrium. Distal occlusion below the common atrium includes the hepatic veins in the Fontan circulation and limits the development of pulmonary arteriovenous malformations, however, it is more difficult to achieve and carries the potential risk of migration into the atrium. Moreover, it is possible to induce the development of other hepatic veins^[1]^. In the first intervention in the current case, venous pressure was 17 mmHg after the occlusion test and systemic oxygen saturation was elevated, so we decided to occlude the fistula from the proximal part. Unfortunately, however, the patient's saturation levels dropped continuously during follow-up, so another intervention was performed to close the fistula distally.

In routine practice, various techniques using gooseneck snares and bioptomes are used to retrieve embolized occluders. Snaring may be challenging at times due to difficulties in catching the retention screw, and the possibility of device distortion is always present. Moreover, potential damage to the surrounding structures is a major concern. Surgical retrieval is probably safe in some of these situations. Re-screwing through wider diameter sheaths is difficult due to problems with approximation between the retention screw and the delivery cable. A secondary sheath with a smaller internal diameter might make it difficult to approximate the retention screw; however, advancing through a larger lumen primary sheath that has been placed close to the device can easily approximate the retention screw^[7]^.

The course of a veno-venous fistula can sometimes be tortuous, causing kinking of the long sheath and not allowing the device to pass through the sheath. Tofeig et al.^[6]^ overcame this problem by front-loading the device into a 7F sheath and delivering it through a 10F long sheath. In a recent report by Kuo^[3]^, a veno-venous fistula was closed via left transhepatic access. The femoral approach is also not suitable in most cases, due to the sharp angle between the inferior vena cava and the hepatic fistula. In our case, we preferred the transjugular approach using a kink-resistant hydrophilic long sheath.

## CONCLUSION

In conclusion, unexpected desaturation after a Fontan operation in patients with heterotaxy should prompt suspicion of the presence of an intrahepatic veno-venous fistula that developed from an accessory hepatic vein connected to the common atrium. Distal transcatheter occlusion of intrahepatic veno-venous fistulas might lead to better clinical outcomes in selected patients. ASO device can be retrieved without any complication within three weeks.

**Table:** 

Authors' roles & responsibilities	
AG	Conception and design, manuscript writing or critical review of its content; final manuscript approval
ICT	Operations and/or experiments performance; manuscript writing or critical review of its content; final manuscript approval
MS	Manuscript writing or critical review of its content; final manuscript approval
